# MicroRNA-Dependent Control of Sensory Neuron Function Regulates Posture Behavior in *Drosophila*

**DOI:** 10.1523/JNEUROSCI.0081-21.2021

**Published:** 2021-10-06

**Authors:** Marleen Klann, A. Raouf Issa, Sofia Pinho, Claudio R. Alonso

**Affiliations:** Sussex Neuroscience, School of Life Sciences, University of Sussex, Brighton BN1 9QG, United Kingdom

## Abstract

All what we see, touch, hear, taste, or smell must first be detected by the sensory elements of our nervous system. Sensory neurons, therefore, represent a critical component in all neural circuits and their correct function is essential for the generation of behavior and adaptation to the environment. Here, we report that the evolutionarily-conserved microRNA (miRNA) *miR-263b* plays a key behavioral role in *Drosophila melanogaster* through effects on the function of larval sensory neurons. Several independent experiments (in 50:50 male:female populations) support this finding: first, miRNA expression analysis, via reporter expression and fluorescent-activated cell sorting (FACS)-quantitative PCR (qPCR) analysis, demonstrate *miR-263b* expression in larval sensory neurons. Second, behavioral tests in *miR-263b* null mutants show defects in self-righting, an innate and evolutionarily conserved posture-control behavior that allows larvae to rectify their position if turned upside-down. Third, competitive inhibition of *miR-263b* in sensory neurons using a *miR-263b* “sponge” leads to self-righting defects. Fourth, systematic analysis of sensory neurons in *miR-263b* mutants shows no detectable morphologic defects in their stereotypic pattern, while genetically-encoded calcium sensors expressed in the sensory domain reveal a reduction in neural activity in *miR-263b* mutants. Fifth, *miR-263b* null mutants show reduced “touch-response” behavior and a compromised response to sound, both characteristic of larval sensory deficits. Furthermore, bioinformatic miRNA target analysis, gene expression assays, and behavioral phenocopy experiments suggest that *miR-263b* might exert its effects, at least in part, through repression of the basic helix-loop-helix (bHLH) transcription factor *Atonal*. Altogether, our study suggests a model in which miRNA-dependent control of transcription factor expression affects sensory function and behavior.

**SIGNIFICANCE STATEMENT** Sensory neurons are key to neural circuit function, but how these neurons acquire their specific properties is not well understood. Here, we examine this problem, focusing on the roles played by microRNAs (miRNAs). Using *Drosophila*, we demonstrate that the evolutionarily-conserved miRNA *miR-263b* controls sensory neuron function allowing the animal to perform an adaptive, elaborate three-dimensional movement. Our work thus shows that microRNAs can control complex motor behaviors by modulating sensory neuron physiology, and suggests that similar miRNA-dependent mechanisms may operate in other species. The work contributes to advance the understanding of the molecular basis of behavior and the biological roles of microRNAs within the nervous system.

## Introduction

Although mechanisms of biological communication are fundamental to biological processes across all scales and species (Lerner et al., 2016), the nervous system is probably one of the best examples of high-speed and complex information transmitted within a cellular network.

In neural systems, input information is represented by sensory signals which provide the brain with essential information about the external environment, so that adequate actions can be selected and implemented. Sensory information is encoded in the form of firing patterns of populations of peripheral neurons, collectively known as sensory neurons. To recognise minor, yet potentially crucial, changes in the external world, sensory neurons must be able to detect diverse stimuli, and transform and transmit this information to the rest of the system. This requires that neural network components are “tuned” or aligned by biochemical machineries operating through a common language, so that neurons can talk to one another in an efficient manner, at rates compatible with the speed of the behaviors they control. Several genetic systems modulate neuronal biochemistries, including dynamic quantitative feed-back control devices able to monitor concentrations of gene products and maintain them within a suitable range. These molecular control devices rely on both, transcriptional as well as posttranscriptional processes.

In this article, we focus on the posttranscriptional component, studying the roles played by small regulatory non-coding RNAs termed microRNAs (miRNAs) on the specification of sensory neurons in *Drosophila* larvae. miRNAs regulate the expression of suites of protein encoding mRNAs by inducing their degradation and/or blocking their translation into protein (Bartel, 2018). Removal of miRNA genes can lead to target de-repression (upregulation), and this may, in certain circumstances, disrupt neural functions critical to physiology and behavioral control.

Our focus on miRNA roles is based on our recent discovery that mutation of a single miRNA gene in *Drosophila* disrupts a particular larval locomotor behavior termed self-righting: a movement that restores normal position after the animal is placed upside-down (Picao-Osorio et al., 2015). Mapping the “focus” of action (Benzer, 1967; Hotta and Benzer, 1972) of this miRNA (*miR-iab4*) led to the finding that this gene did not affect neural development, and instead, controls the physiology of a specific set of motor neurons in the larva (Picao-Osorio et al., 2015). Extensions of this work demonstrated that *miR-iab4* also controls self-righting in the *Drosophila* adult (Issa et al., 2019) through actions on a different set of motor neurons, indicating that individual miRNAs may affect equivalent behaviors on systems bearing profoundly different neuroanatomy and biomechanics. Furthermore, a genetic screen (Picao-Osorio et al., 2017) aimed at identifying all miRNAs with impact on larval self-righting, revealed a pervasive influence of miRNA control on this postural behavior.

Self-righting is a complex evolutionarily conserved, three-dimensional, adaptive, and innate locomotor sequence (Ashe, 1970; Faisal and Matheson, 2001; Jusufi et al., 2011). To trigger this movement, the fruit fly larva must, first, determine that its position is abnormal. This suggests that sensory processes may play a key role in this behavior. *Drosophila* larvae possess a wide range of sensory organs including multidendritic (md) sensory neurons (SNs) (Bodmer and Jan, 1987; Grueber et al., 2002) and chordotonal organs (Field and Matheson, 1998) capable of detecting chemical and mechanical inputs. These sensory systems are present on the body wall, arranged in highly stereotypical patterns within each segment of the larva, and show complex and largely invariant morphologies (Zawarzin, 1912; Hartenstein, 1988).

Here, we show that the evolutionarily conserved miRNA *miR-263b* is essential for larval self-righting, through modulatory effects on the sensory system. Cell ablation experiments show that sensory neurons are essential for self-righting, and gene expression analyses demonstrate that *miR-263b* is expressed across different populations of larval sensory neurons. In addition, genetic manipulations show that normal *miR-263b* expression in these sensory elements is essential for normal self-righting. Morphologic analyses demonstrate that lack of *miR-263b* does not disrupt the intricate morphology or array of larval sensory neurons, suggesting miRNA impact on neural function, rather than on structure. Behavioral and optical imaging experiments confirm this, indicating that the functionality and physiology of sensory neurons is abnormal in the absence of *miR-263b*. Lastly, based on bioinformatic, gene expression and behavioral analyses, we propose a model in which *miR-263b* exerts its actions on sensory neurons via repression of the basic helix-loop-helix (bHLH) transcription factor encoded by the gene *atonal*. Our work thus provides evidence that miRNAs can control complex motor behaviors by modulating the physiology of sensory neurons.

## Materials and Methods

### *Drosophila melanogaster* strains

Cultures of *D. melanogaster* stocks were kept under standard conditions with 50–60% relative humidity and a 12/12 h light/dark cycle at 18°C, while working copies were held at 25°C. The following stocks were used in this study: 109(2)80-Gal4 (BDSC #8769), iav-Gal4 (BDSC #52273), ppk-1.9-Gal4 (stock described in Ainsley et al., 2003; a gift from Matthias Landgraf), ato-Gal4 (BDSC # 6480), 109(2)80-Gal4,UAS-mCD8::GFP (BDSC #8768) ΔmiR-263b-Gal4 (stock described in Hilgers et al., 2010; a gift from Sherry Aw), UAS-TeTN Lc (BDSC #28838), UAS-nls-GFP (BDSC #4775), UAS-miR-263b sponge (BDSC #61403), *UAS-scramble sponge* (BDSC #61501), UAS-GCaMP6m (BDSC #42748), Δ*miR-263b* (BDSC #58903), UAS-ato (BDSC #39679); *w^1118^* flies (BDSC #5905) were used as controls.

### Immunohistochemistry

Embryo collection, dechorionation, devitellinization, and fixation were conducted as previously described (Picao-Osorio et al., 2015) and samples were stored in 100% methanol at −20°C. To obtain various embryonic stages an overnight collection was used. The samples were gradually rehydrated, rinsed in 1× PBS and 0.3% Triton X-100 (PBTx) and washed with PBTx 4 × 30 min. Subsequently, the samples were incubated with primary antibody (in PBTx) over night at 4°C. Primary antibodies and their concentration used are: 1:10 mouse anti-22C10 (Developmental Studies Hybridoma Bank), 1:2000 chicken anti-GFP (Abacam Probes), 1:100 guinea pig anti-atonal (a gift from Daniel Marenda) and goat anti-HRP-A556. Primary antibody was removed with three rinses and 4 × 30 min PBTx washes. The secondary antibody was added and incubated for 2 h at room temperature. Secondary antibodies and their concentrations used are: 1:500 anti-mouse Alexa Fluor 488 (Invitrogen), 1:500 anti-mouse Alexa Fluor A555 (Invitrogen), 1:500 anti-chicken Alexa Fluor 488 (Invitrogen), 1:500 anti-guinea pig Alexa Fluor A555 or A488. During the incubation with the secondary antibody 1:500–1:1000 Hoechst 33 342 (Life Technologies) was added. Samples were rinsed three times, washed 4 × 30 min with PBTx and transferred to 75% glycerol for mounting. Samples were imaged on a Leica SP8 confocal laser scanning microscope and further processed using ImageJ/FIJI and Adobe Photoshop CS6. Schematic representations and figure arrangement were made with Adobe Illustrator CS6.

### *In vivo* calcium imaging

The calcium sensor GCaMP6m was used to measure the Ca^2+^ signal in SNs. Calcium imaging was conducted on stage 17 embryos to reduce movement during the recording. Parental lines were raised at 25°C in collection cages bearing apple juice-based medium agar plates, supplemented with yeast paste. Before recording, stage 16 embryos were collected, dechorionated and transferred into a drop (1 µl) of PBS (to prevent dehydration) previously left on a poly-L-lysine coated glass slides. The embryo was placed with its lateral side up, to allow visibility of the majority of SNs. Next, the Ca^2+^ signal within the SNs was captured during 3 min using Leica DM6000 microscope (Leica Microsystems) and processed with the software Fiji imageJ. GCaMP signals from the soma were analysed. The average signal from the first 10-s was taken as fluorescence baseline F_0_ to calculate the ΔF/F_0_ for each recording.

### Cell sorting experiments

The fluorescent-activated cell sorting (FACS) dissociation protocol followed was the one described by Harzer and colleagues (Harzer et al., 2013) with some adjustments. Control (w^1118^) and experimental larvae [109(2)80-Gal4,UAS-mCD8::GFP; 50 h old] were opened anterodorsally and the gut was partly removed. For each genotype larvae were dissected for 30 min, usually obtaining 50–60 larvae, which were pooled in an Eppendorf tube filled with Rinaldini's solution (800 mg NaCl, 20 mg KCl, 5 mg NaH_2_PO_4_, 100 mg NaHCO_3_, and 100 mg glucose in 100 ml H_2_O). The larvae were washed once with 500-µl Rinaldini's solution. Rinaldini's solution was replaced with dissociation buffer, the mixture was incubated for 1 h at 30°C and gently mixed twice during incubation. Dissociation buffer needs to be prepared fresh, by adding 25-µl collagenase Type I (20 mg/ml, Sigma-Aldrich) and 25-µl papain (20 mg/ml, Sigma-Aldrich) to 200-µl complete Schneider's culture medium (5 ml heat-inactivated fetal bovine serum, 0.1 ml insulin, 1 ml PenStrep, 5 ml L-glutamine, 0.4 ml L-glutathione, and 37.85 ml Schneider's medium). All subsequent washing steps need to be conducted very slowly and carefully to avoid premature dissociation of the larval bodies. After the removal of the dissociation solution, the samples were washed twice with 500-µl Rinaldini's solution first, and then twice with 500-µl complete Schneider's culture medium. All medium was removed and the larval bodies were dissociated using 200-µl complete Schneider's medium, which was pipetted up and down (medium and larvae) with as little foaming as possible. The solution started to appear homogenous after pipetting was repeated ∼50 times. The cell suspension was filtered through a 30-µm mesh into a 5-ml FACS tube, placed on ice, and immediately taken to a FACS system (BD-FACSMelody). One biological replicate comprised around 3000 sorted cells (one sorting event), cells were sorted directly into TRIzol (Invitrogen) and stored at −80°C until further processing.

### Quantitative PCR (qPCR) assays

A standard phenol/chloroform protocol was employed to extract RNA from FACS samples using Phase Lock Gel 2 ml Heavy tubes (Fisher Scientific). RNA was treated for 30 min with DNase (TURBO DNA-free, Invitrogen) subsequently. SuperScript III First Strand (Invitrogen) was used for cDNA synthesis. A single qPCR is made of 5 µl 2xSYBR green mix (LightCycler 480 SYBR Green I master, Roche), 2 µl H_2_O, 1 µl 5 μm forward primer, 1 µl 5 μm reverse primer, 1 µl cDNA (diluted 1:2 with H_2_O). Every sample was run in technical triplicates. The cycle conditions used were 40× (10 s 95°C, 20 s 60°C, 20 s 72°C) with fluorescent readings during annealing and elongation on a QuantStudio 3 machine (Thermo Fisher Scientific). Three reference genes were tested, Cdc5, RpS9, and eEF1α, and Cdc5 was chosen as appropriate reference gene. Primer sequences used are listed below:*miR-263b* (forward) 5′-ACTTTGAGTCTTGGCACTGG-3′*miR-263b* (reverse) 5′-GAAATCGTTGTACAAAGCCGG-3′*miR-10* (forward) 5′-GCTTGCCATCAGCAACACTTT-3′*miR-10* (reverse) 5′-CGGACTTCATTTCGCCCCAG-3′

*GFP* (forward) 5′-CATTCATCAGCCGTCTTCCG-3′

*GFP* (reverse) 5′-GAGTGCCCAAGAAAGCTACC-3′*futsch* (forward) 5′-TATTAGGGAAGACGCCGACC-3′*futsch* (reverse) 5′-AGGACTGGAGGCCTTAATGC-3′

*Cdc5* (forward) 5′-CGGCAAGATCGAGAAGAAGC-3′

*Cdc5* (reverse) 5′-GTTCTGCTCAATCTGGCCG-3′

For each primer set a standard curve was generated using serial dilution of the cDNA (undiluted, 1:2, 1:4, 1:8, 1:16, and 1:32), which was employed to calculate primer efficiency E (E = 10^(−1/slope)^). The primer efficiency was incorporated into the formula to calculate fold change values (R) as described below:

$$R=\frac{E{\left(geneofinterest\right)}^{\Delta Ctgeneofinterest(w1118-experimental)}}{E{\left(referencegene\right)}^{\Delta Ctreferencegene(w1118-experimental)}}$$

### Sequences, statistics, and miRNA target prediction

Sequences for *miR-263b/183/228* were recovered from miRBase.org (release 22) for schematic representation of the phylogenetic tree. Statistical analysis was done using Microsoft Excel. Statistical significance was calculated using Student's *t* tests with a *p* value threshold of 0.05 for significance. For *atonal* expression, fluorescent signal comparisons from wild-type and Δ*miR-263b* mutant embryos were processed the same/at the same time and images were collected using the same settings. ImageJ was employed to analyse fluorescent intensity of individual *atonal*-positive clusters, with the help of the region of interest (ROI) manager. A ROI was manually drawn around an *atonal*-positive cell cluster as well as a region adjacent to the *atonal*-positive clusters where *atonal* is not expressed (to normalize background expression). This was repeated for a minimum of seven clusters (14 cluster-pairs), all located in the thoracic or abdominal region of the embryo. After all ROIs were selected, they were measured using “Measure” button within the ROI manager tool. Per ROI four values related to fluorescence were provided by the program: area, mean, minimum and maximum. First, mean intensity was calculated per area (intensity/area). To calculate the fluorescent intensity of an *atonal*-positive cluster, the intensity/area of the background expression (*atonal*-negative area) was subtracted from the intensity/area of the *atonal*-positive cluster. Per embryo between 7 and 10 *atonal*-positive clusters were measured and the average was taken. At least 10 embryos were analysed per genotype. For *D. melanogaster miR-263b* target prediction, we used PITA (Kertesz et al., 2007) and TargetScanFly 7.2 (Agarwal et al., 2018).

### Behavioral tests

All embryos/larvae were kept on apple juice agar plates containing a small amount of yeast paste at 25°C. Self-righting and anterior touch response assays were conducted on freshly hatched L1 larvae (<30 min old). For those, late-stage embryos were transferred to a fresh apple juice agar plate (without yeast paste) and monitored for emerging larvae. Newly hatched L1 larvae were transferred to another fresh apple juice agar plate, which was used for testing throughout one session (1 biological repeat). Early L3 larvae (72 h) were used for the sound response assay (startle assay). At least three biological replicates with a minimum of 20 larvae per replicate were analysed for each behavioral test, including self-righting, touch-response, and sound response/startle. A paint brush was used to roll larvae for self-righting tests, which were otherwise performed as described in (Picao-Osorio et al., 2015). An eyelash was employed to deliver a soft stroke to the anterior region (head and thorax only) for anterior touch response assays. The protocol followed those described by Kernan and colleagues (Kernan et al., 1994). Overall response score and hesitation time were analysed. For sound response/startle assays the protocol developed by Zhang and colleagues (Zhang et al., 2013) was followed. In brief, 72 h larvae were washed with PBS and transferred to a testing plate. The plate was put on top of a speaker and videotaped from above. Larval response to sound was assayed by stimulation with a 1-s sound pulse (pure tone, 400 Hz), which was repeated 10 times.

### Experimental design and statistical analysis

Time to self-righting, sound response, hesitation time, and atonal fluorescence intensity graphs were generated and compared using GraphPad Prism 6 software. Sample size of *n* ≥ 50 and *n* = 6–10 animals were used in total per genotype for behavioral and functional/imaging assays, respectively; each experiment was performed at least three times. Mean and SEM values were calculated for each trial and analysed by Mann–Whitney test, or one-way ANOVA and Kruskal–Wallis tests, when more groups were present (as indicated in each figure). GraphPad Prism 6 was used for all statistical analyses. Significant values in all figures: *p < 0.05, **p < 0.01, ***p < 0.001.

## Results

### Sensory and genetic requirements for self-righting behavior

**Figure 1.**
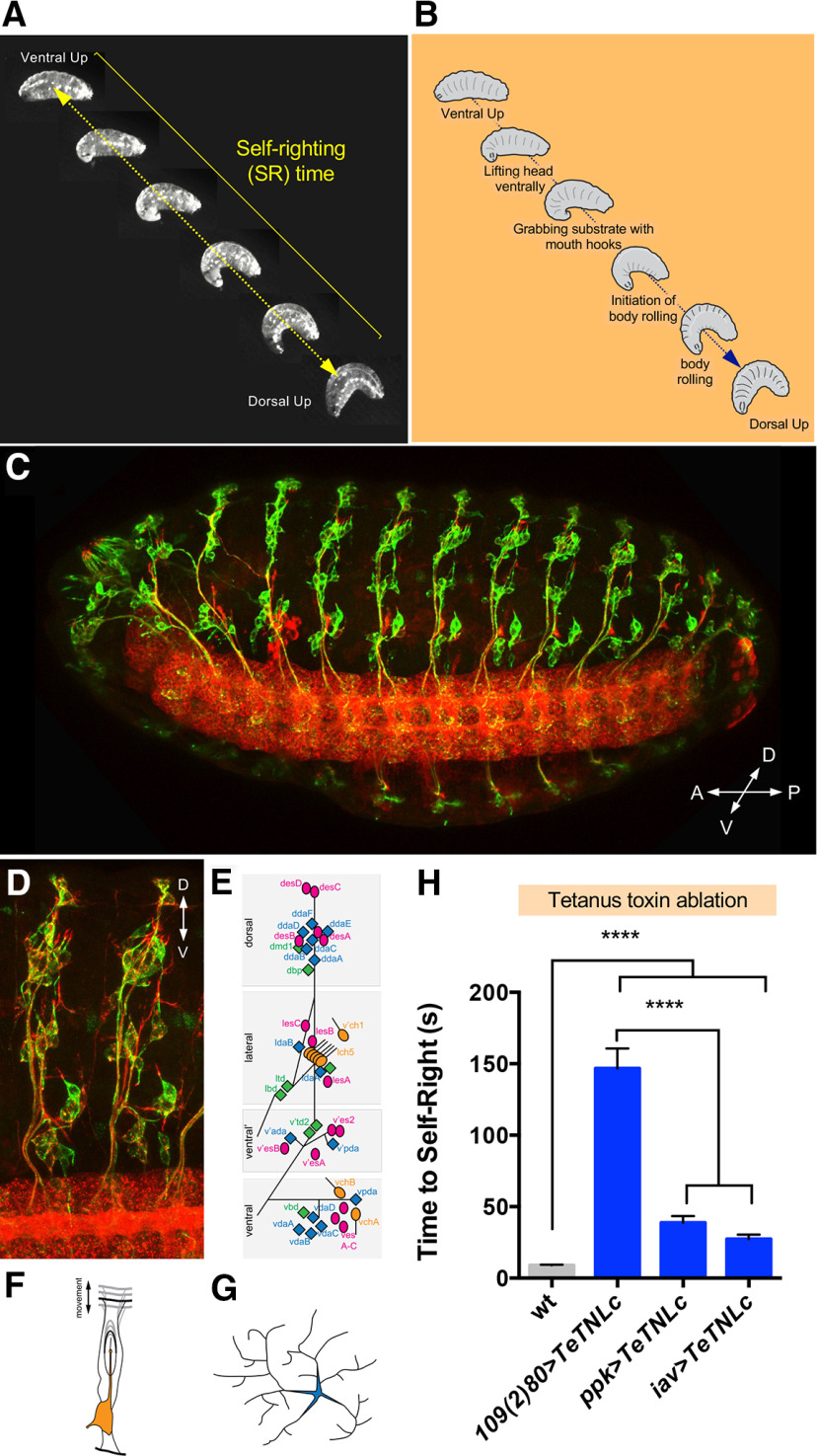
Self-righting behavior and its sensory requirements. ***A***, The self-righting sequence shown as a series of still images acquired from a video captured during larval self-righting (arrow, self-righting time). ***B***, Self-righting sequence. Schematic representation indicating the individual steps observed during the self-righting behavioral sequence. ***C***, The sensory system of stage 16 *Drosophila* embryo, in ventro-lateral view. Sensory neurons were labeled with α-22C10 [green; α-HRP (red) provides a general neuronal marker]. ***D***, Sensory organ arrangement of embryonic abdominal segments, ventro-lateral view. Note the complexity and regularity of the arrangement of elements. Schematic representation of the distribution and composition of embryonic/larval sensory organs in the abdomen (***E***), morphology of chordotonal organs (***F***), and md neurons (***G***) is depicted by means of cartoons. ***H***, Self-righting performance is decreased when all components of the sensory system (109(2)80) or some of them (ppk, md SNs; iav, chordotonal organs) are disabled by the tetanus toxin, indicating that information detected by the sensory system is essential for normal larval self-righting (*n* > 60, ANOVA and Kruskal–Wallis test).

The self-righting response is an innate and evolutionarily conserved movement that involves body rotation when the organism is placed upside-down (Ashe, 1970; Faisal and Matheson, 2001; Jusufi et al., 2011; Picao-Osorio et al., 2015). In *Drosophila* larvae, self-righting concerns the coordinated three-dimensional motion of multiple larval segments (Fig. 1*A*,*B*) and is triggered by the inversion of the position of the larval body in respect to the substrate. A possibility to explain the activation of self-righting is that the response might be triggered by a change in the orientation of the gravitational field; but this is not the case: inversion of the polarity of the gravitational field does not affect larval movement or trigger the self-righting response (Movies 1, 2). An alternative model is that the animal detects an anomaly in the normal pattern of sensory stimuli that informs the status of its contact with substrate and, based on this change, triggers the self-righting sequence. Peripheral sensory inputs are detected by genetically-defined subsets of larval sensory neurons arranged dorsoventrally along the larval body wall (Zawarzin, 1912; Hartenstein, 1988), including md sensory neurons (Bodmer and Jan, 1987; Grueber et al., 2002) and chordotonal organs (Field and Matheson, 1998; Fig. 1*C–E*). We hypothesised that these peripheral sensors may play a role in conveying the necessary information that triggers self-righting behavior, and, to test this model, we disabled subsets of peripheral SNs using the tetanus toxin (Sweeney et al., 1995) and examined the effects of these perturbations on self-righting. The results of these experiments demonstrate that SNs demarked by expression of the drivers *109(2)80* (multiple dendritic neurons, oenocytes, and chordotonal organs; Gao et al., 1999), *ppk* (Class IV dendritic arborization neurons and, less strongly, Class III neurons; Grueber et al., 2002, 2007), and *iav* (chordotonal neurons; Kwon et al., 2010) are essential for normal self-righting (Fig. 1*F*).

Movie 1. Substrate exploration of a *Drosophila* first instar larva under normal gravitational conditions. The movie shows substrate exploratory behavior of a wild-type *Drosophila* first instar larva (∼30 min old) when the gravitational field is in a normal orientation (right-side up). Note that during the sequence, the specimen displays normal forward locomotion and turning behavior, but does not engage in self-righting behavior (Fig. 1). 10.1523/JNEUROSCI.0081-21.2021.video.1

Movie 2. Substrate exploration of a *Drosophila* first instar larva under inverted gravitational conditions. The movie shows substrate exploratory behavior of a wild-type *Drosophila* first instar larva (∼30 min old) when the gravitational field is in an inverted orientation (wrong-side up). Note that, just as in when the experiment is conducted under a normal orientation of the gravitational field, the specimen displays normal forward locomotion and turning behavior but does not engage in self-righting behavior (Fig. 1). These observations demonstrate that an inversion of the polarity of the gravitational field is insufficient to trigger self-righting behavior, indicating that *Drosophila* larvae must utilize other sensory information than gravity, to trigger the self-righting sequence. 10.1523/JNEUROSCI.0081-21.2021.video.2

### Genetic elements affecting self-righting sensory function: a role for small non-coding RNAs

**Figure 2.**
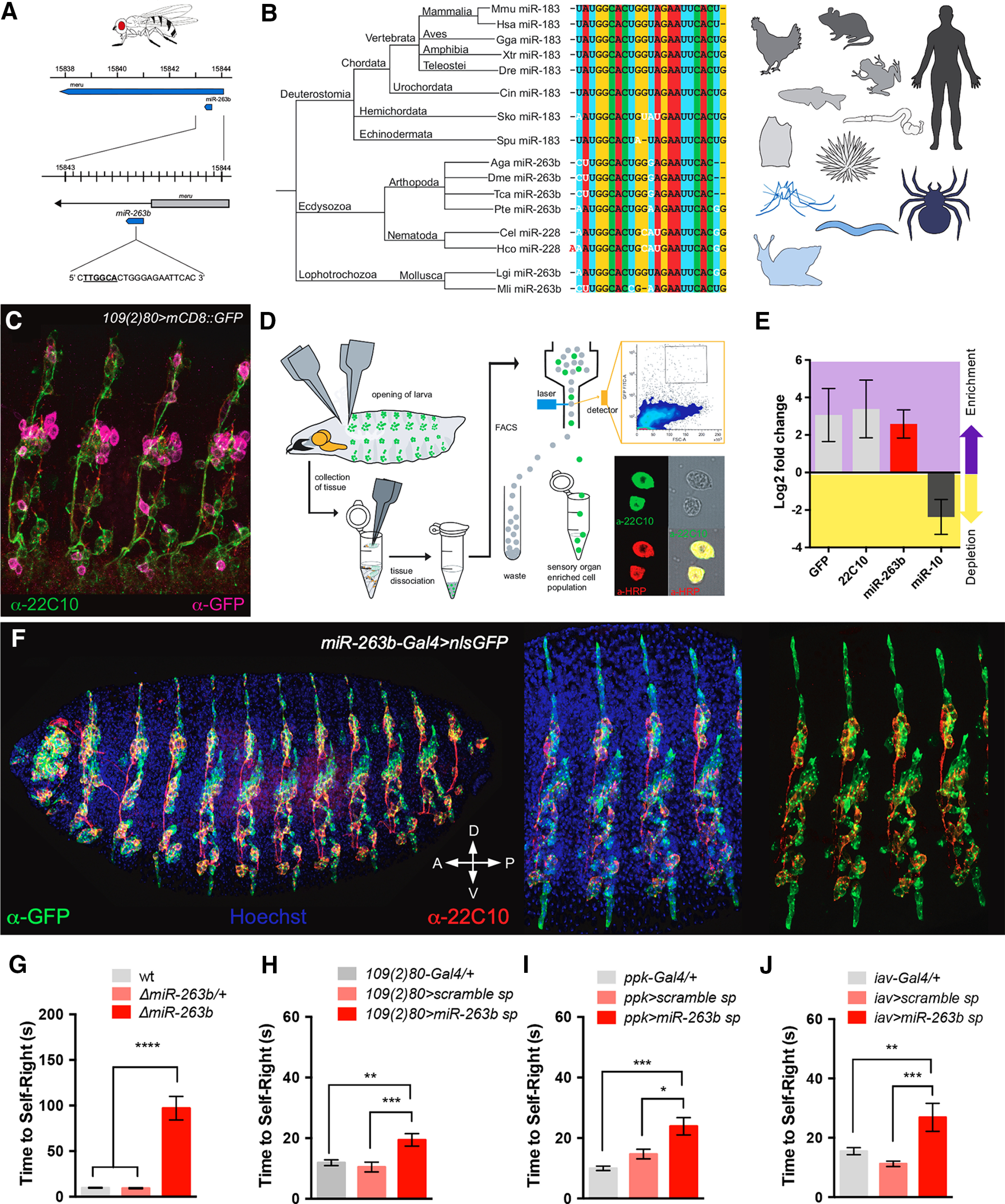
Expression and behavioral roles of *miR-263b*. ***A***, Genomic location of *miR-263b* in *Drosophila*. ***B***, Evolutionary conservation of *miR-263b* throughout the animal kingdom. The sequence encoding *miR-263b* is highly conserved across large phylogenetic distances; there are several names for this miRNA: *miR-263b* in arthropods and molluscs; *miR-183* in deuterostomes; *miR-228* in nematodes. ***C***, The driver line *109(2)80-Gal4* was used in cell sorting (FACS) experiments; its coupling to the *UAS-mCD8::GFP* construct labels larval sensory neurons (magenta). ***D***, Schematic representation of the FACS protocol used to study miRNA expression in the sensory system. The process involved the dissection of larval tissue (top left), and cell type validation via antibody staining (bottom right). ***E***, Relative gene expression of GFP and *futsch* (labeled as “22C10”) in sensory organ enriched cell populations. ***F***, *miR-263b* expression using a the *miR-263b* driver confirms miRNA expression in sensory organ-associated cells in the *Drosophila* embryo. Sensory neurons are visualized in red (α-22C10) and expression of the *miR-263b* driver is depicted via GFP signal, in green (α-GFP). ***G***, *miR-263b* homozygote mutants (red) exhibit a pronounced delay in self-righting. ***H***, Reduction of *miR-263b* function via competitive inhibition (miRNA sponges) in md neurons and chordotonal neurons results in a very significant increase of self-righting time. ***I***, Functional inhibition of *miR-263b* in md (Class IV) neurons, or in (***J***) chordotonal organs, leads to significant delays in self-righting time (for panels ***G–J***, we used sample size *n* > 50, ANOVA and Kruskal–Wallis tests). Altogether these results indicate that normal expression of *miR-263b* in the sensory system is necessary for normal larval behavior.

The physiology of SNs (and that of all neurons) largely relies on biochemistry; and the latter, is defined by the gene expression programs active in the cell. In this context, genetic elements with regulatory roles, i.e., affecting the expression of cohorts of target genes, might play an important function in setting the global biochemical and physiological properties of neurons, allowing for specific contributions to behavior. We have recently identified several genes encoding small non-coding RNAs, i.e., miRNAs, that are essential for a normal self-righting response in *Drosophila* (Picao-Osorio et al., 2015, 2017; Issa et al., 2019). Several of these miRNAs, collectively termed SR-miRNAs (self-righting miRNAs; Picao-Osorio et al., 2017), are expressed in the central nervous system, but others have been previously reported with expression in the peripheral nervous system (PNS) of larvae and adult (Pierce et al., 2008; Clark et al., 2010; Hilgers et al., 2010; Sun et al., 2012). Among these, we became particularly interested in *miR-263b* (Pierce et al., 2008; Hilgers et al., 2010) because of its pervasive evolutionary conservation (Pierce et al., 2008; Fig. 2*A*,*B*) and roles in multiple sensory and neural processes in the adult (Hilgers et al., 2010; Nian et al., 2019) and set to determine whether *miR-263b* might play a direct role in the SNs underlying self-righting behavior. For this we first aimed at establishing the expression pattern of *miR-263b* in larval SNs. FACS experiments coupled to qPCR expression assays (Fig. 2*D*,*E*), demonstrate expression of *miR-263b* in Drosophila larval SNs. Furthermore, spatial expression analysis of a Gal4 insertion located within the *miR-263b* locus (Hilgers et al., 2010) confirms expression of this genetic element within SNs in the embryo (Fig. 2*F*) providing independent experimental evidence that supports expression of *miR-263b* in SNs involved in self-righting control.

The fact that *miR-263b* is expressed in larval SNs is consistent with a potential role of this miRNA in SNs, but falls short from demonstrating functional roles. A more direct way to test the involvement of *miR-263b* in the biology of SNs is genetic removal: if *miR-263b* is essential for SN function, elimination of this genetic element from larvae is predicted to affect behaviors that rely on normal SNs, including self-righting. Experiments in Figure 2*G* show that homozygote as *miR-263b* mutant larvae display severe self-righting phenotypes demonstrating that this miRNA gene is essential for normal self-righting. Furthermore, functional attenuation of *miR-263b* by means of miRNA-specific sponges (Fulga et al., 2015) applied to the whole sensory domain or within specific sensory subsets (Fig. 2*H–J*) further confirms that *miR-263b*-mediated activities are necessary for normal self-righting behavior to take place.

### *miR-263b* affects the physiology of sensory neurons

**Figure 3.**
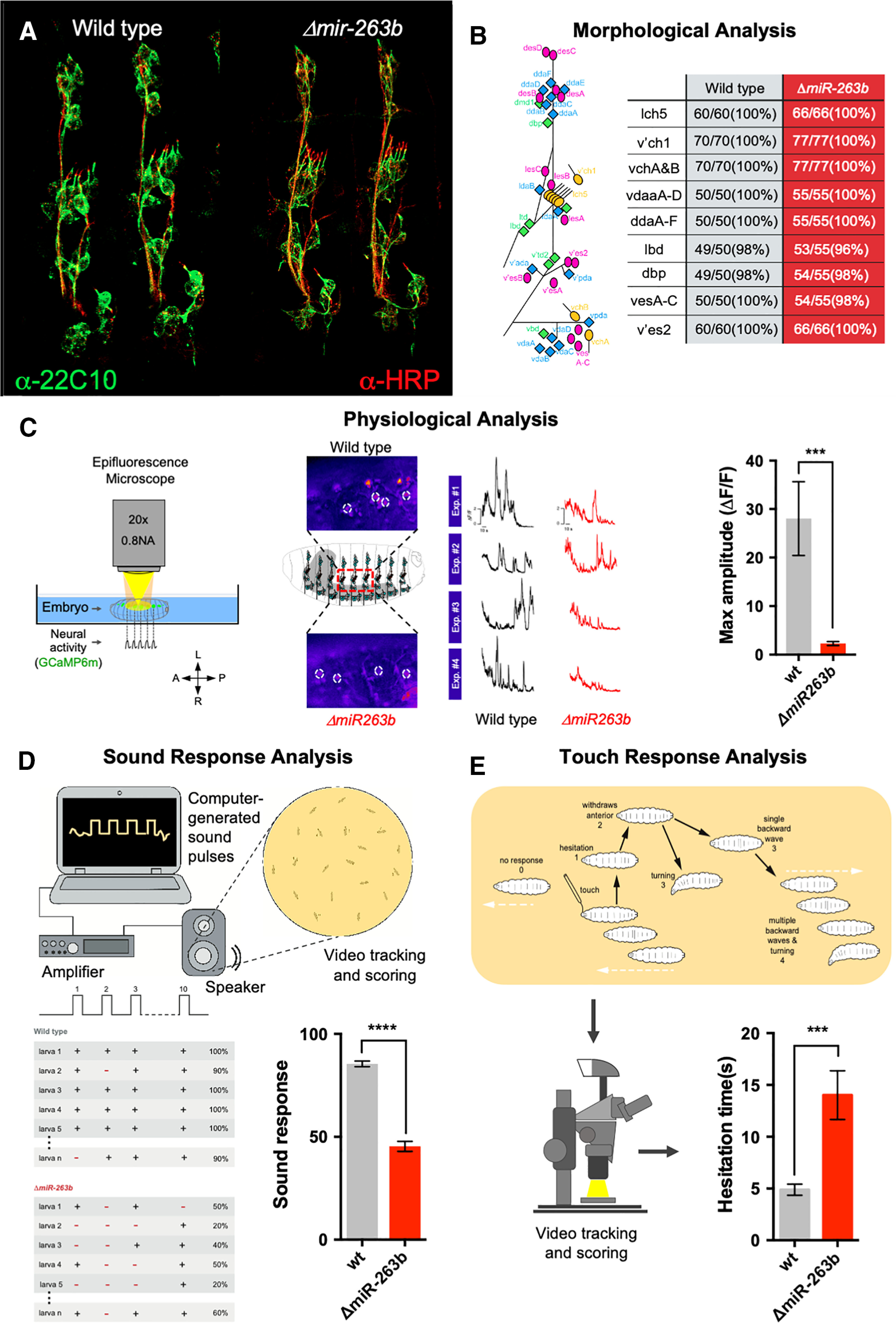
Effects of *miR-263b* on neural morphology, physiology and behavior. ***A***, Labeling of sensory neurons (green, α-22C10) in stage 16 embryos in wild-type and *miR-263b* mutant specimens reveals the detailed morphology of the sensory system. ***B***, Comparison of the integrity and arrangements of a comprehensive series of sensory organ subtypes (see diagram on the left) in wild-type and *miR-263b* mutants; there are no morphologic anomalies detected in *miR-263b* mutants. ***C***, Calcium sensor analysis (GCaMP6m) shows reduced neural activity in *miR-263b* mutants when compared with wild type (*n* = 6, Mann–Whitney test). ***D***, Experimental set up for sound response experiments (top). Diagram describing a representative set of sound response experiments in both genotypes (bottom left); quantification of responsiveness to sound (bottom right) is reduced in *miR-236b* mutants (*n* > 50, Mann–Whitney test). ***E***, Experimental setup and scoring system for anterior touch-response experiments; video tracking and quantification of individual responses in normal and miRNA mutant larvae shows that *miR-236b* mutants exhibit an increased hesitation time (bottom right; *n* > 60, Mann–Whitney test). These experiments suggest that *miR-263b* plays a role in the physiological control of sensory function with impact on behavior.

The regulatory nature of miRNAs makes them suitable for regulation of multiple functions within the organism, including developmental as well as physiological roles. To examine the point of action of *miR-263b* in the sensory system, we first set to establish whether this miRNA controls the development of SNs. For this we looked at the morphology of the sensory system in *miR-263b* null mutant larvae and wild-type specimens. The complex and stereotyped morphology of the larval sensory field makes it particularly suitable as a system to examine effects on the developmental process. Figure 3*A*,*B* shows that detailed and systematic characterization of the individual sub-components of the sensory system using confocal microscopy, in both, wild-type and *miR-263b* null mutant larvae, reveals no detectable differences among these two genotypes (Fig. 3*B*).

The absence of morphological defects in the sensory system of *miR-263b* null mutants might indicate that this miRNA may impinge on the function (physiology) rather than the development of SNs. To test this possibility directly, we decided to quantify the spontaneous patterns of neural activity produced by SNs in miRNA mutants and compare these results to those obtained in normal specimens. For this we expressed a genetically-encoded calcium sensor (GCaMP6m) within the sensory system and visualized the resulting patterns of activity in live recordings. Figure 3*C* shows the results of these experiments where a significant decrease in neural activity patterns can be observed in *miR-263b* samples (Fig. 3*C*).

We reasoned that if *miR-263b* affects the general physiology of SNs, then other larval behaviors that rely on sensory input could also be affected in the absence of this miRNA. Two independent series of experiments confirm this. First, quantitative assessment of larval response to sound, previously shown to depend on sensory neurons, particularly, on chordotonal organs (Zhang et al., 2013), reveal that *miR-263b* mutants display a marked decrease in sound responsiveness when compared with their wild-type counterparts (Fig. 3*D*). Second, evaluation of the ability of larva to respond to touch (“anterior touch-response”; Kernan et al., 1994) shows that *miR-263b* mutants display a reduced response with markedly pronounced “hesitation time” (Fig. 3*G*,*H*). Altogether, the combination of morphologic, physiological and behavioral analyses of *miR-263b* mutants strongly indicates that this miRNA is required for normal sensory function in *Drosophila* larvae.

### A molecular model for *miR-263b* action in sensory neurons and self-righting behavior

**Figure 4.**
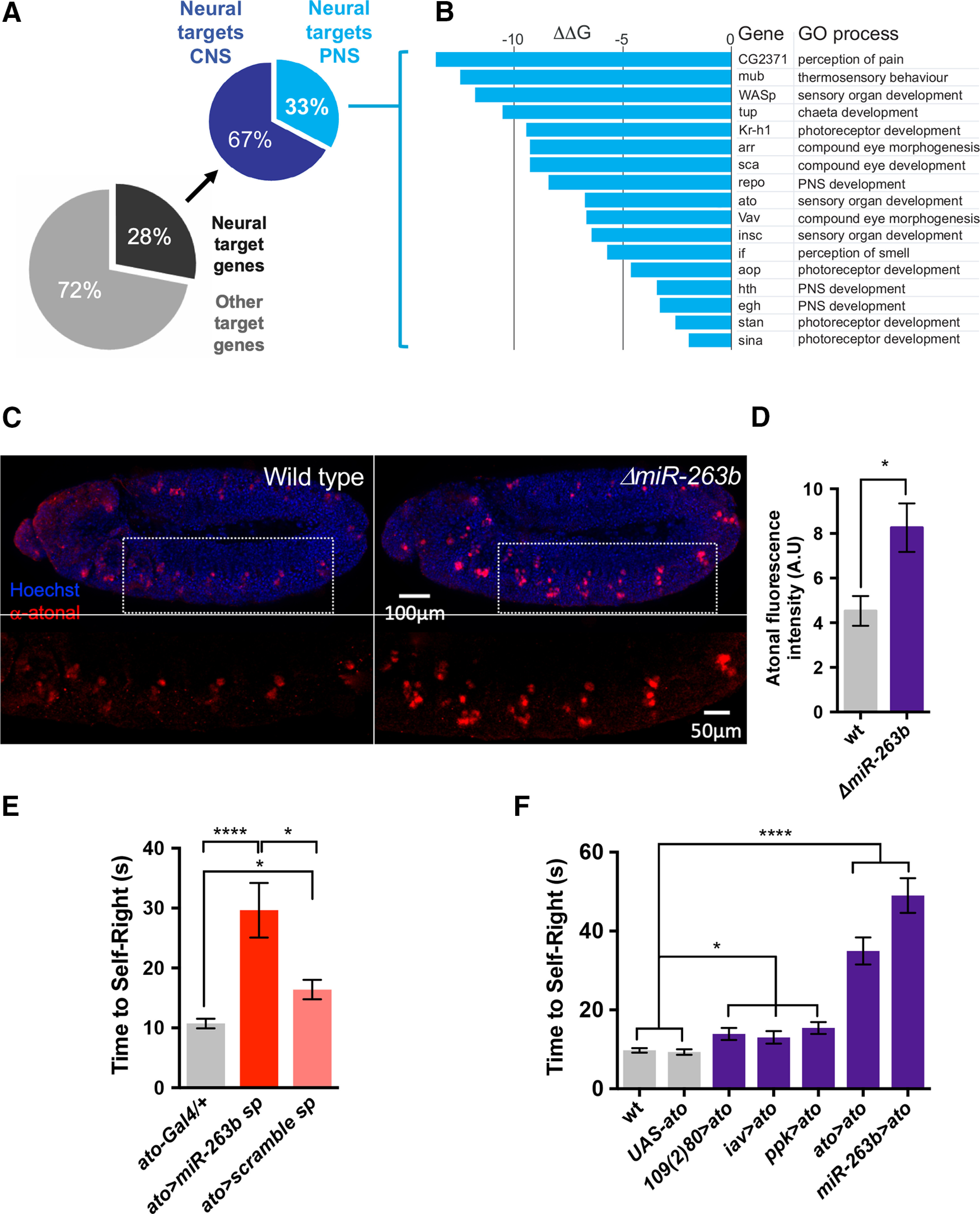
Potential molecular targets of *miR-263b* and their roles in self-righting. ***A***, Pie charts representing shared predicted target genes of *miR-263b* through the combined output of the PITA and TargetScan algorithms. Out of 186 total miRNA targets highlighted by both computational approaches, 52 (28%) can be classified (GO terms) as “neuronal targets”; within this group, 17 relate to the PNS. ***B***, Details on the 17 predicted target genes involved in the PNS, note the proneural gene *atonal* (*ato*) within the top-ten predicted target genes of *miR-263b* [NB: expression/process associations are according to Gene Ontology (GO) analysis]. ***C***, Atonal protein expression analysis in wild-type and *miR-263b* mutant specimens reveals that Atonal expression is elevated in *miR-263b* mutants. ***D***, Quantification of antibody labeling experiments for Atonal demonstrate that the levels of this proneural protein are significantly increased in *miR-263b* mutants (*n* = 10, Mann–Whitney test). ***E***, Reduction of *miR-263b* function within the *atonal* expression domain increases self-righting time (*n* > 60, Mann–Whitney test). ***F***, Artificial increase of *atonal* expression (i) within different sensory fields (activated via *109(2)80*, *ppk*, and *iav* drivers); (ii) within the endogenous *atonal* expression domain; or (iii) within the *miR-263b* transcriptional domain all result in significantly increased self-righting times (*n* > 60, ANOVA and Kruskal–Wallis test). These data suggest that atonal might be one of the molecular targets that mediates the roles of *miR-263b* in the sensory system.

To advance the mechanistic understanding of the effects of *miR-263b* in the sensory system we decided to explore the potential points of action of this miRNA within SNs. Given that miRNAs are regulatory molecules (Bartel, 2018) their biological roles in the cell are likely to emerge indirectly, via effects on so-called “miRNA target” genes. Bioinformatic prediction of miRNA targets for *miR-263b* using the PITA and TargetScan algorithms (Kertesz et al., 2007; Agarwal et al., 2018) indicate a considerable number (*n* = 186) of potential targets of this miRNA in the *Drosophila* transcriptome highlighted simultaneously by both algorithms; approximately one-third of these targets (28%) show expression in the nervous system (Fig. 4*A*). Within the predicted set of neural targets, 33% have demonstrated expression in the PNS. Among the top-ten predicted *miR-263b* molecular targets with PNS expression, most genes encode factors with known functions in eye morphogenesis and differentiation, including: *WASp* (Wiskott–Aldrich syndrome protein; Ben-Yaacov et al., 2001), *tup* (tailup, also known as islet; Thor and Thomas, 1997), *Kr-h1* (Krüppel homolog 1; Fichelson et al., 2012), *arr* (arrow; Wehrli et al., 2000), *sca* (scabrous; Mlodzik et al., 1990), *repo* (reverse polarity; Xiong et al., 1994), *Vav* (Vav guanine nucleotide exchange factor; Malartre et al., 2010), and the bHLH transcription factor Atonal encoded by the *atonal* (*ato*) gene. The latter was of particular interest to us because of its role as a proneural gene during the specification and development of chordotonal organs (Jarman et al., 1993; Jarman and Ahmed, 1998), among other sensory organs (Jarman et al., 1995; Gupta and Rodrigues, 1997). Chordotonal organs are internal stretch receptors that detect cuticle movement and vibration, and have been shown to be important for detecting sound (Zhang et al., 2013). As we expect chordotonal organs to play roles in all three behaviors tested (i.e., self-righting, sound-response and touch-response), we selected *atonal* as an entry point for detailed functional studies.

Three independent series of experiments support the model that *atonal* is one of the factors that mediates the actions of *miR-263b* in the sensory system with effects on self-righting control. First, *atonal* is expressed in sensory neurons, and its expression is increased in miRNA null mutants, in line with the expected de-repression effect caused by removal of a repressive regulatory miRNA (Fig. 4*C*,*D*). Second, expression of a *miR-263b-sponge* within the *atonal* domain is sufficient to trigger a self-righting phenotype (Fig. 4*E*). Third, artificial upregulation of *atonal* within its natural transcriptional domain in wild-type larvae, so that its expression level emulates those observed in *miR-263b* mutants, is sufficient to phenocopy the self-righting defect observed in miRNA mutants (Fig. 4*F*); similar effects are observed when *ato* is specifically delivered within a sub-field of the sensory system or when driven within the transcriptional domain of *miR-263b* (Fig. 4*F*).

**Figure 5.**
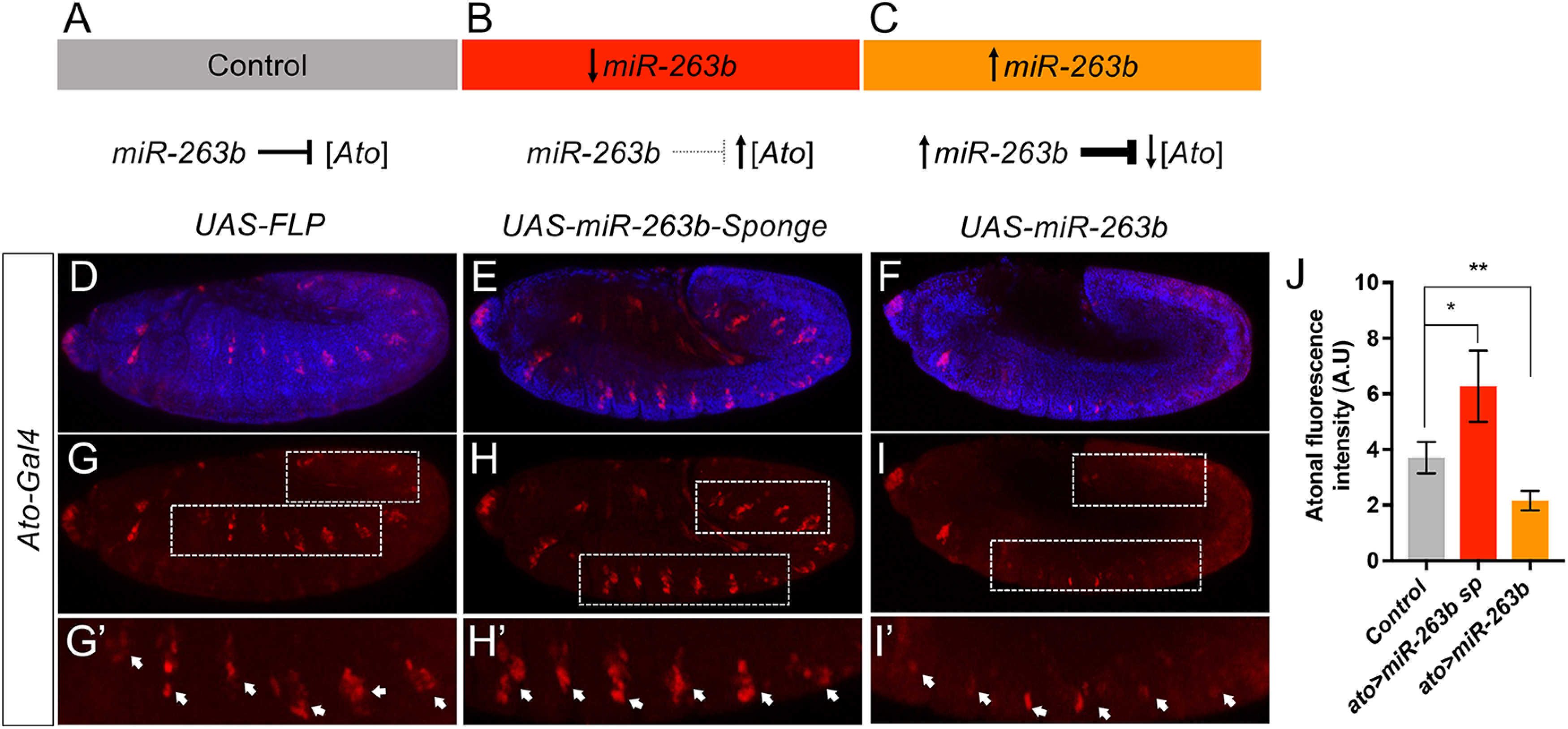
Regulatory effects of *miR-263b* on the expression of the bHLH transcription factor Atonal. ***A***–***C***, Predicted regulatory effects of *miR-263b* on atonal expression. ***A***, According to our regulatory model, in control conditions, *miR-263b* is expected to repress the expression of *atonal*, contributing to the maintenance of concentration values of Atonal protein within a physiological range compatible with normal sensory neuron function. ***B***, Reduction of *miR-263b* function is expected to de-repress *atonal* expression, leading to an increase in Atonal protein concentration. ***C***, In contrast, upregulation of *miR-263b* expression is predicted to reduce the levels of expression of Atonal below normal concentration. ***D–F***, Experimental validation of the predicted regulatory effects of *miR-263b* on Atonal expression within the natural *atonal* transcriptional domain (*Ato-Gal4*). ***D***, ***G***, ***G'***, Normal expression of Atonal (red) as detected by immunohistochemistry using anti-Atonal antibodies. ***E***, ***H***, ***H'***, Functional reduction of *miR-263b* within the *atonal* expression domain by means of expressing a *miR-263b-sponge* leads to an increase in Atonal signal (red), in line with predicted de-repression effects (see ***B***). ***F***, ***I***, ***I'***, Increase of *miR-263b* expression leads to downregulation of Atonal expression (red), as predicted by the *miR-263b*-dependent Atonal expression control model, see ***C*** (blue signal: DAPI). ***J***, Quantification of the regulatory interactions between *miR-263b* and Atonal shows a statistically significant increase of Atonal expression when *miR-263b* function is reduced (*ato*>*miR-263b-sp*), and reduction of Atonal signal when *miR-263b* is overexpressed (*ato*>*miR-263b*). These observations validate the proposed *miR-263b*-dependent regulatory model for atonal expression control in sensory neurons (*n* ≥ 13, Mann–Whitney test).

We next decided to further probe the relation between *miR-263b* and *atonal*. For this, we conducted a series of experiments in which we artificially increased (UAS-*miR-263b*) or decreased (UAS-*miR-263b* sponge) the function of *miR-263b* within the natural *atonal* expression domain: should there be a direct regulatory link between *miR-263b* and *atonal* genes, an increment of miRNA repression is predicted to reduce Atonal protein signal, while a reduction of miRNA function should lead to Atonal upregulation (Fig. 5*A–C*). The data shown in Figure 5*D–J* provide experimental validation of both these predictions. Expression of *miR-263b* leads to a significant decrease in atonal expression (Fig. 5*E*,*H*,*H'*), while expression of *miR-263b-sp* induces an increase of Atonal signal (Fig. 5*F*,*I*,*I'*). These data support a regulatory interaction between *miR-263b* and *atonal* within neurons.

Together, these results suggest a model by which *miR-263b*-mediated repression of *atonal* in sensory neurons is essential for a normal self-righting response.

## Discussion

We have employed *Drosophila* larvae to investigate the sensory elements required for triggering an evolutionarily-conserved 3-D postural behavior (self-righting) and established that normal expression of a miRNA gene, *miR-263b*, in the larval sensory system is necessary for a normal self-righting response. Our experiments also explore the causal links between the absence of *miR-263b* and the resulting behavioral effects, and show that absence of *miR-263b* does not seem to affect the complex morphologic organisation of sensory neurons in the fruit fly larvae. Instead, we observe an impairment in several sensory functions (touch-response, sound detection) as well as a reduced level of spontaneous neural activity in the sensory neurons of *miR-263b* mutants. Based on these results we suggest that *miR-263b* is required for the normal physiological control of sensory function in *Drosophila* larvae, with no detectable effects in the formation of the sensory system. At the mechanistic level, the combination of bioinformatic, gene expression and behavioral analyses indicate that *miR-263b* might exert its actions, at least in part, through repression of the bHLH transcription factor *atonal*, a regulatory proneural gene with known roles in the formation of sensory elements in *Drosophila* (Jarman et al., 1993, 1995; Bossuyt et al., 2009) and mammals (Bermingham et al., 1999; Zheng and Gao, 2000).

Evidence from behavioral genetic studies across different species indicates that the genetics of behavior is often complex and that the path between individual genes and behavioral traits is frequently intricate and hard to work out (Greenspan, 2008). Here, we see a different picture, in which reduction in the expression of a miRNA gene (caused by either a single loss-of-function mutation or tissue-specific functional attenuation) leads to a consistent behavioral change. Yet, how exactly a reduced level of expression of *miR-263b* modifies the properties of sensory elements in the fruit fly maggot, causing a change in behavior, remains open. In this respect, our observations suggest that *miR-263b* absence leads to an increase in the expression of *atonal*, which, instead of derailing the developmental process, affects the biochemistry and physiology of sensory cells.

Indeed, experiments in rat explants indicate that an excess of *atonal*'s orthologue in the rat (Math1) can promote the formation of hair cells out of a population of postnatal utricular supporting cells (Zheng and Gao, 2000); this shows that going above a particular “set-point” of Atonal protein concentration (as observed in our study) is sufficient to modify the cellular processes in sensory elements. Given that *atonal* is expressed in *Drosophila* chordotonal organs (Jarman et al., 1993; Jarman and Ahmed, 1998) as well as in the cochlear system of mammals (Bermingham et al., 1999; Yang et al., 2012; Jarman and Groves, 2013), it might be plausible that *atonal*-dependent events could impact on behavior in other systems too. Interestingly, in the mouse, expression of Math5 and NeuroD1 (both, murine orthologues of *atonal*) is detected in postmitotic cells in the retina, consistent with a postdevelopmental role of *atonal* in mammalian sensory neurons (Yang et al., 2003); in particular, *NeuroD* expression has been shown to play a role in the terminal differentiation of retinal neurons in mammals (Ahmad et al., 1998). Also, in *Caenorhabditis elegans*, *hlh4*, a bHLH transcription factor related to the *Atonal/Achaete-Scute complex* class, has been shown to be required for the normal postdevelopmental function of nociceptive neurons (Masoudi et al., 2018). More broadly, gene expression analysis of vertebrate and invertebrate proneural proteins, including those encoded by atonal orthologues, shows signal in postmitotic neurons, where these proteins are believed to regulate neuronal migration and axonal/dendritic growth (Guillemot and Hassan, 2017).

A previous investigation in our laboratory (Picao-Osorio et al., 2015) showed that a deficit of another miRNA, *miR-iab4*, causes a change in the physiology of larval motor neurons involved in self-righting. In this case, *miR-iab4* mediates the repression of the Hox protein Ultrabithorax (Ubx), a homeodomain-containing transcription factor (Bridges and Morgan, 1923; Sánchez-Herrero et al., 1985; Mallo and Alonso, 2013); in addition, an increase of *Ubx* phenocopies the behavioral effects observed in *miR-iab4* mutants (Picao-Osorio et al., 2015).

Building on our findings on *miR-263b* and *miR-iab4*, we propose the model that miRNA-dependent control of behavior relies on maintaining the expression levels of a small set of transcription factors (*ato*, *Ubx*) within a particular concentration range; departures from such range may lead to gene regulatory effects with impact on neuronal biochemistry, which in ultimate instance, modify the physiological properties of the cell. This model, however, may be at odds with current views on the molecular mechanisms of miRNA function, which strongly indicate that miRNAs regulate multiple targets within the cell (Bartel, 2018; McGeary et al., 2019) arguing that regulation via single or few target genes is unlikely. A potential framework that reconciles both scenarios is that for specific cells (i.e., neurons) miRNA-dependent regulation of just a small subset of targets (*ato*, *Ubx*) is critical for their biological function, while other regulatory events are simply tolerated, or compensated for, by the regulatory networks of the cell (Alonso, 2012) and/or the neural circuits underlying behavior. Observations of miRNA effects on simple nematode behaviors (CO_2_ response) seem also to rely on the modulation of a few target genes (Drexel et al., 2016), suggesting that the generality of our proposed model might extend to other animal phyla.

It should also be noted, that self-righting was disrupted in ∼40% of miRNA mutants tested in an earlier study in our lab (Picao-Osorio et al., 2017) suggesting that many defects caused by miRNAs can affect self-righting, in addition to the above-mentioned cases of *miR-iab4* and *miR-263b*. Indeed, while *miR-263b* mutants often appear to delay efforts to self-right (in line with their sensory deficits), some other miRNA mutants seem simply “sluggish,” or display sequences of unproductive peristaltic waves and head twists (Picao-Osorio et al., 2017); these observations suggest that each miRNA may affect self-righting in a particular way. Investigation of how this comprehensive set of “self-righting miRNAs” affects behavior is currently under way in our laboratory, and, once completed, should allow a more thorough testing of the model suggested above.

Our results show that ablation of either all, or genetically-defined subtypes of sensory elements in the larvae lead to a significant impairment in self-righting, but how sensory information is transformed into the actual self-righting response in the larva is still unknown. A current effort (Picao-Osorio, O'Garro-Priddie, Cardona and Alonso, in preparation) is using neural connectomics and reconstruction at synaptic resolution to map the neural substrates of self-righting behavior and should, when completed, offer a cellular platform for the investigation of how information in the sensory system is transmitted and converted into the motor patterns that underlie the self-righting sequence.

Previous work showed that *miR-263b* plays an important role in the *Drosophila* PNS, where it forms part of a gene regulatory pathway that represses apoptosis during sense organ development (Hilgers et al., 2010), and other studies report that downregulation or mutation of *miR-263b* lead to distortions of adult locomotion patterns (Donelson et al., 2020) or affect stereotypical parameters of male–female courtship (Iftikhar et al., 2019), respectively. Further studies in the adult indicate that *miR-263b* is also involved in the regulation of circadian behaviors (Nian et al., 2019; You et al., 2018). Our work adds to these studies, suggesting that *miR-263b* underlies a wide-spectrum of neural functions across developmental stages, implying the likely existence of positive selective pressure to retain a functional version of this gene in the *Drosophila* genome. Indeed, we note that *miR-263b* is evolutionary conserved within the Drosophilids, but also across long taxonomical distances. Furthermore, regarding the *miR-263b* gene family, and in addition to its close relative miRNA *miR-183* (Fig. 2*B*), we wish to point out that the evolutionarily conserved *miR-96* has an identical “seed” to *miR-263b* suggesting similar specificity (Pierce et al., 2008); intriguingly, *miR-96* is required for normal hearing in mice and humans (Krohs et al., 2021; Lewis et al., 2009).

Building on the extensive phylogenetic preservation of *miR-263b* and its relative miRNAs, and the similarly broad conservation of *atonal* (Quan and Hassan, 2005; Cai and Groves, 2015) and that of self-righting itself (Ashe, 1970; Faisal and Matheson, 2001; Jusufi et al., 2011), we speculate that *miR-263b* and *atonal* orthologues in other species may play a role in self-righting and other postural control mechanisms in other orders of animals. Although the idea that the same sets of miRNAs and targets may contribute to establish the cellular biochemistry that enables similar behavioral responses in animals with drastically distinct body plans (e.g., insects and mammals), may seem unlikely, a recent observation on miRNA function in our lab does lend support to this possibility: *miR-iab4*-dependent control of *Ubx* expression is required for normal self-righting behavior in both *Drosophila* larvae and adults, two morphs of the fly that bear radically different morphologies, neural constitution and biomechanics (Issa et al., 2019).

Our study shows that the function of sensory neurons may rely on the normal expression of small non-coding RNAs, and that this is relevant for a complex and adaptive behavioral sequence in *Drosophila* larvae illustrating that behavior emerges from a careful balance in the expression of transcriptional as well as posttranscriptional gene regulators within the nervous system.

## References

[B1] Agarwal V, Subtelny AO, Thiru P, Ulitsky I, Bartel DP (2018) Predicting microRNA targeting efficacy in *Drosophila*. Genome Biol 19:152. 10.1186/s13059-018-1504-3 30286781 PMC6172730

[B2] Ahmad I, Acharya HR, Rogers JA, Shibata A, Smithgall TE, Dooley CM (1998) The role of NeuroD as a differentiation factor in the mammalian retina. J Mol Neurosci 11:165–178. 10.1385/JMN:11:2:165 10096043

[B3] Ainsley JA, Pettus JM, Bosenko D, Gerstein CE, Zinkevich N, Anderson MG, Adams CM, Welsh MJ, Johnson WA (2003) Enhanced locomotion caused by loss of the *Drosophila* DEG/ENaC protein Pickpocket1. Curr Biol 13:1557–1563. 10.1016/S0960-9822(03)00596-7 12956960

[B4] Alonso CR (2012) A complex 'mRNA degradation code' controls gene expression during animal development. Trends Genet 28:78–88. 10.1016/j.tig.2011.10.005 22257633

[B5] Ashe MV (1970) The righting reflex in turtles: a description and comparison. Psychon Sci 20:150–152. 10.3758/BF03335647

[B6] Bartel DP (2018) Metazoan MicroRNAs. Cell 173:20–51. 10.1016/j.cell.2018.03.006 29570994 PMC6091663

[B7] Ben-Yaacov S, Le Borgne R, Abramson I, Schweisguth F, Schejter ED (2001) Wasp, the *Drosophila* Wiskott-Aldrich syndrome gene homologue, is required for cell fate decisions mediated by Notch signaling. J Cell Biol 152:1–13. 10.1083/jcb.152.1.1 11149916 PMC2193661

[B8] Benzer S (1967) Behavioral mutants of *Drosophila* isolated by countercurrent distribution. Proc Natl Acad Sci USA 58:1112–1119. 10.1073/pnas.58.3.1112 16578662 PMC335755

[B9] Bermingham NA, Hassan BA, Price SD, Vollrath MA, Ben-Arie N, Eatock RA, Bellen HJ, Lysakowski A, Zoghbi HY (1999) Math1: an essential gene for the generation of inner ear hair cells. Science 284:1837–1841. 10.1126/science.284.5421.1837 10364557

[B10] Bodmer R, Jan YN (1987) Morphological differentiation of the embryonic peripheral neurons in *Drosophila*. Rouxs Arch Dev Biol 196:69–77. 10.1007/BF00402027 28305460

[B11] Bossuyt W, De Geest N, Aerts S, Leenaerts I, Marynen P, Hassan BA (2009) The atonal proneural transcription factor links differentiation and tumor formation in *Drosophila*. PLoS Biol 7:e40. 10.1371/journal.pbio.1000040 19243220 PMC2652389

[B12] Bridges CB, Morgan TH (1923) The third-chromosome group of mutant characters of Drosophila melanogaster. Washington, DC: Carnegie Institution of Washington.

[B13] Cai T, Groves AK (2015) The role of atonal factors in mechanosensory cell specification and function. Mol Neurobiol 52:1315–1329. 10.1007/s12035-014-8925-0 25339580 PMC4587357

[B14] Clark AM, Goldstein LD, Tevlin M, Tavaré S, Shaham S, Miska EA (2010) The microRNA miR-124 controls gene expression in the sensory nervous system of *Caenorhabditis elegans*. Nucleic Acids Res 38:3780–3793. 10.1093/nar/gkq083 20176573 PMC2887956

[B15] Donelson NC, Dixit R, Pichardo-Casas I, Chiu EY, Ohman RT, Slawson JB, Klein M, Fulga TA, Van Vactor D, Griffith LC (2020) MicroRNAs regulate multiple aspects of locomotor behavior in *Drosophila*. G3 (Bethesda) 10:43–55. 10.1534/g3.119.400793 31694853 PMC6945011

[B16] Drexel T, Mahofsky K, Latham R, Zimmer M, Cochella L (2016) Neuron type-specific miRNA represses two broadly expressed genes to modulate an avoidance behavior in *C. elegans*. Genes Dev 30:2042–2047. 27688400 10.1101/gad.287904.116 PMC5066611

[B17] Faisal AA, Matheson T (2001) Coordinated righting behaviour in locusts. J Exp Biol 204:637–648. 10.1242/jeb.204.4.637 11171346

[B18] Fichelson P, Brigui A, Pichaud F (2012) Orthodenticle and Kruppel homolog 1 regulate *Drosophila* photoreceptor maturation. Proc Natl Acad Sci USA 109:7893–7898. 10.1073/pnas.1120276109 22547825 PMC3356647

[B19] Field LH, Matheson T (1998) Chordotonal organs of insects. Adv Insect Physiol 28:1–228.

[B20] Fulga TA, McNeill EM, Binari R, Yelick J, Blanche A, Booker M, Steinkraus BR, Schnall-Levin M, Zhao Y, DeLuca T, Bejarano F, Han Z, Lai EC, Wall DP, Perrimon N, Van Vactor D (2015) A transgenic resource for conditional competitive inhibition of conserved *Drosophila* microRNAs. Nat Commun 6:7279. 10.1038/ncomms8279 26081261 PMC4471878

[B21] Gao FB, Brenman JE, Jan LY, Jan YN (1999) Genes regulating dendritic outgrowth, branching, and routing in *Drosophila*. Genes Dev 13:2549–2561. 10.1101/gad.13.19.2549 10521399 PMC317067

[B22] Greenspan RJ (2008) Seymour Benzer (1921-2007). Curr Biol 18:R106–R110. 10.1016/j.cub.2007.12.039 18345547

[B23] Grueber WB, Jan LY, Jan YN (2002) Tiling of the *Drosophila* epidermis by multidendritic sensory neurons. Development 129:2867–2878. 10.1242/dev.129.12.2867 12050135

[B24] Grueber WB, Ye B, Yang CH, Younger S, Borden K, Jan LY, Jan YN (2007) Projections of *Drosophila* multidendritic neurons in the central nervous system: links with peripheral dendrite morphology. Development 134:55–64. 10.1242/dev.02666 17164414

[B25] Guillemot F, Hassan BA (2017) Beyond proneural: emerging functions and regulations of proneural proteins. Curr Opin Neurobiol 42:93–101. 10.1016/j.conb.2016.11.011 28025176

[B26] Gupta BP, Rodrigues V (1997) Atonal is a proneural gene for a subset of olfactory sense organs in *Drosophila*. Genes Cells 2:225–233. 10.1046/j.1365-2443.1997.d01-312.x 9189759

[B27] Hartenstein V (1988) Development of *Drosophila* larval sensory organs: spatiotemporal pattern of sensory neurones, peripheral axonal pathways and sensilla differentiation. Development 102:869–886. 10.1242/dev.102.4.869

[B28] Harzer H, Berger C, Conder R, Schmauss G, Knoblich JA (2013) FACS purification of *Drosophila* larval neuroblasts for next-generation sequencing. Nat Protoc 8:1088–1099. 10.1038/nprot.2013.062 23660757 PMC3930877

[B29] Hilgers V, Bushati N, Cohen SM (2010) *Drosophila* microRNAs 263a/b confer robustness during development by protecting nascent sense organs from apoptosis. PLoS Biol 8:e1000396. 10.1371/journal.pbio.1000396 20563308 PMC2885982

[B30] Hotta Y, Benzer S (1972) Mapping of behaviour in *Drosophila* mosaics. Nature 240:527–535. 10.1038/240527a0 4568399

[B31] Iftikhar H, Johnson NL, Marlatt ML, Carney GE (2019) The role of miRNAs in *Drosophila melanogaster*. Male Courtship Behavior. Genetics 211:925–942. 10.1534/genetics.118.301901 30683757 PMC6404249

[B32] Issa AR, Picao-Osorio J, Rito N, Chiappe ME, Alonso CR (2019) A single microRNA-Hox gene module controls equivalent movements in biomechanically distinct forms of *Drosophila*. Curr Biol 29:2665–2675.e4. 10.1016/j.cub.2019.06.082 31327720 PMC6710004

[B33] Jarman AP, Ahmed I (1998) The specificity of proneural genes in determining *Drosophila* sense organ identity. Mech Dev 76:117–125. 10.1016/S0925-4773(98)00116-6 9767145

[B34] Jarman AP, Groves AK (2013) The role of Atonal transcription factors in the development of mechanosensitive cells. Semin Cell Dev Biol 24:438–447. 10.1016/j.semcdb.2013.03.010 23548731 PMC3778674

[B35] Jarman AP, Grau Y, Jan LY, Jan YN (1993) atonal is a proneural gene that directs chordotonal organ formation in the *Drosophila* peripheral nervous system. Cell 73:1307–1321. 10.1016/0092-8674(93)90358-W 8324823

[B36] Jarman AP, Sun Y, Jan LY, Jan YN (1995) Role of the proneural gene, atonal, in formation of *Drosophila* chordotonal organs and photoreceptors. Development 121:2019–2030. 10.1242/dev.121.7.2019 7635049

[B37] Jusufi A, Zeng Y, Full RJ, Dudley R (2011) Aerial righting reflexes in flightless animals. Integr Comp Biol 51:937–943. 10.1093/icb/icr114 21930662

[B38] Kernan M, Cowan D, Zuker C (1994) Genetic dissection of mechanosensory transduction: mechanoreception-defective mutations of *Drosophila*. Neuron 12:1195–1206. 10.1016/0896-6273(94)90437-5 8011334

[B39] Kertesz M, Iovino N, Unnerstall U, Gaul U, Segal E (2007) The role of site accessibility in microRNA target recognition. Nat Genet 39:1278–1284. 10.1038/ng2135 17893677

[B40] Krohs C, Körber C, Ebbers L, Altaf F, Hollje G, Hoppe S, Dörflinger Y, Prosser HM, Nothwang HG (2021) Loss of miR-183/96 alters synaptic strength via presynaptic and postsynaptic mechanisms at a central synapse. J Neurosci 41:6796–6811. 10.1523/JNEUROSCI.0139-20.2021 34193555 PMC8360680

[B41] Kwon Y, Shen WL, Shim HS, Montell C (2010) Fine thermotactic discrimination between the optimal and slightly cooler temperatures via a TRPV channel in chordotonal neurons. J Neurosci 30:10465–10471. 10.1523/JNEUROSCI.1631-10.2010 20685989 PMC3335392

[B42] Lerner TN, Ye L, Deisseroth K (2016) Communication in neural circuits: tools, opportunities, and challenges. Cell 164:1136–1150. 10.1016/j.cell.2016.02.027 26967281 PMC5725393

[B43] Lewis MA, Quint E, Glazier AM, Fuchs H, De Angelis MH, Langford C, van Dongen S, Abreu-Goodger C, Piipari M, Redshaw N, Dalmay T, Moreno-Pelayo MA, Enright AJ, Steel KP (2009) An ENU-induced mutation of miR-96 associated with progressive hearing loss in mice. Nat Genet 41:614–618. 10.1038/ng.369 19363478 PMC2705913

[B44] Malartre M, Ayaz D, Amador FF, Martín-Bermudo MD (2010) The guanine exchange factor vav controls axon growth and guidance during *Drosophila* development. J Neurosci 30:2257–2267. 10.1523/JNEUROSCI.1820-09.2010 20147552 PMC6634040

[B45] Mallo M, Alonso CR (2013) The regulation of Hox gene expression during animal development. Development 140:3951–3963. 10.1242/dev.068346 24046316

[B46] Masoudi N, Tavazoie S, Glenwinkel L, Ryu L, Kim K, Hobert O (2018) Unconventional function of an Achaete-Scute homolog as a terminal selector of nociceptive neuron identity. PLoS Biol 16:e2004979. 10.1371/journal.pbio.2004979 29672507 PMC5908064

[B47] McGeary SE, Lin KS, Shi CY, Pham TM, Bisaria N, Kelley GM, Bartel DP (2019) The biochemical basis of microRNA targeting efficacy. Science 366:eaav1741. 10.1126/science.aav1741 31806698 PMC7051167

[B48] Mlodzik M, Baker NE, Rubin GM (1990) Isolation and expression of scabrous, a gene regulating neurogenesis in *Drosophila*. Genes Dev 4:1848–1861. 10.1101/gad.4.11.1848 2125959

[B49] Nian X, Chen W, Bai W, Zhao Z, Zhang Y (2019) miR-263b controls circadian behavior and the structural plasticity of pacemaker neurons by regulating the LIM-only protein Beadex. Cells 8:923. 10.3390/cells8080923 PMC6721658 31426557

[B50] Picao-Osorio J, Johnston J, Landgraf M, Berni J, Alonso CR (2015) MicroRNA-encoded behavior in *Drosophila*. Science 350:815–820. 10.1126/science.aad0217 26494171 PMC4902127

[B51] Picao-Osorio J, Lago-Baldaia I, Patraquim P, Alonso CR (2017) Pervasive behavioral effects of microRNA regulation in *Drosophila*. Genetics 206:1535–1548. 10.1534/genetics.116.195776 28468905 PMC5500149

[B52] Pierce ML, Weston MD, Fritzsch B, Gabel HW, Ruvkun G, Soukup GA (2008) MicroRNA-183 family conservation and ciliated neurosensory organ expression. Evol Dev 10:106–113. 10.1111/j.1525-142X.2007.00217.x 18184361 PMC2637451

[B53] Quan XJ, Hassan BA (2005) From skin to nerve: flies, vertebrates and the first helix. Cell Mol Life Sci 62:2036–2049. 10.1007/s00018-005-5124-1 16003490 PMC11139128

[B54] Sánchez-Herrero E, Vernós I, Marco R, Morata G (1985) Genetic organization of *Drosophila* bithorax complex. Nature 313:108–113. 10.1038/313108a0 3917555

[B55] Sun K, Westholm JO, Tsurudome K, Hagen JW, Lu Y, Kohwi M, Betel D, Gao F-B, Haghighi AP, Doe CQ, Lai EC (2012) Neurophysiological defects and neuronal gene deregulation in *Drosophila* mir-124 mutants. PLoS Genet 8:e1002515. 10.1371/journal.pgen.1002515 22347817 PMC3276548

[B56] Sweeney ST, Broadie K, Keane J, Niemann H, O'Kane CJ (1995) Targeted expression of tetanus toxin light chain in *Drosophila* specifically eliminates synaptic transmission and causes behavioral defects. Neuron 14:341–351. 10.1016/0896-6273(95)90290-2 7857643

[B57] Thor S, Thomas JB (1997) The *Drosophila* islet gene governs axon pathfinding and neurotransmitter identity. Neuron 18:397–409. 10.1016/S0896-6273(00)81241-6 9115734

[B58] Wehrli M, Dougan ST, Caldwell K, O'Keefe L, Schwartz S, Vaizel-Ohayon D, Schejter E, Tomlinson A, DiNardo S (2000) arrow encodes an LDL-receptor-related protein essential for wingless signalling. Nature 407:527–530. 10.1038/35035110 11029006

[B59] Xiong WC, Okano H, Patel NH, Blendy JA, Montell C (1994) repo encodes a glial-specific homeo domain protein required in the *Drosophila* nervous system. Genes Dev 8:981–994. 10.1101/gad.8.8.981 7926782

[B60] Yang SM, Chen W, Guo WW, Jia S, Sun JH, Liu HZ, Young WY, He DZ (2012) Regeneration of stereocilia of hair cells by forced Atoh1 expression in the adult mammalian cochlea. PLoS One 7:e46355. 10.1371/journal.pone.0046355 23029493 PMC3459923

[B61] Yang Z, Ding K, Pan L, Deng M, Gan L (2003) Math5 determines the competence state of retinal ganglion cell progenitors. Dev Biol 264:240–254. 10.1016/j.ydbio.2003.08.005 14623245

[B62] You S, Fulga TA, Van Vactor D, Jackson FR (2018) Regulation of circadian behavior by astroglial microRNAs in *Drosophila*. Genetics 208:1195–1207. 10.1534/genetics.117.300342 29487148 PMC5844331

[B63] Zawarzin A (1912) Histologische Studien uber Insekten. II Das sensible Nervensystem der Aeschnalarven. Z Wess Zool 100:245–286.

[B64] Zhang W, Yan Z, Jan LY, Jan YN (2013) Sound response mediated by the TRP channels NOMPC, NANCHUNG, and INACTIVE in chordotonal organs of *Drosophila* larvae. Proc Natl Acad Sci USA 110:13612–13617. 10.1073/pnas.1312477110 23898199 PMC3746866

[B65] Zheng JL, Gao WQ (2000) Overexpression of Math1 induces robust production of extra hair cells in postnatal rat inner ears. Nat Neurosci 3:580–586. 10.1038/75753 10816314

